# Evaluation of TRMM 3B43 data over the Yangtze River Delta of China

**DOI:** 10.1038/s41598-018-23603-z

**Published:** 2018-03-28

**Authors:** Yueqian Cao, Wu Zhang, Wenjing Wang

**Affiliations:** 1 Key Laboratory for Semi-Arid Climate Change of the Ministry of Education, College of Atmospheric Sciences, Lanzhou University, Lanzhou, 730000 China; 2 Department of Civil and Environmental Engineering, Pratt School of Engineering, Duke University, Durham, NC 27708 USA

## Abstract

Tropical Rainfall Measuring Mission (TRMM) 3B43 rainfall products during the period of 1998~2016 are evaluated via monthly and yearly precipitation data from 56 observational stations over the Yangtze River Delta (YRD). The results are as follows: (1) annual rainfall increases gradually from northwest to southwest and monthly precipitation surges sharply, owing to rainband’s abrupt pushing north caused by the westward and northward extension of Western Pacific Subtropical High, to 200 mm/month in July; (2) only seized by the TRMM, the developing process of ChunAn precipitation center synchronizes with monthly precipitation series that may recycle throughout the year; (3) 3B43 data are inclined to overestimate precipitation but performs the best (relative bias ranging within −10~10%) at most parts of the YRD zone; and its correlation coefficient with observation is 0.88 in annual scale; for monthly scale, CC peaks in March (0.96) and reaches the bottom (0.79) in August; (4) no evident connection between CC and elevation is found in the 3B43 annual rainfall products which estimate precipitation better upon urban land. This paper is of importance in understanding the impacts of complex topography and landcovers on the precipitation assessment in a river delta scale.

## Introduction

Precipitation is a key component of energy balance and water cycle, it also plays a vital role in the formation of regional weather and global climate. Hence precise precipitation data at high spatial and temporal resolution are extremely desirable for multiple research fields, such as monsoon climate, extreme weather and flood prediction^1–5^. Though conventional rain gauges are deemed as standard precipitation measuring apparatus, their spatial limitation of point-based measurements and relatively insufficient networks worldwide determine that they cannot reflect precipitation distribution reliably whereas remote sensing has become main source of accurate and continuous precipitation data, especially satellite can potentially solve this limitation by affording grid products of rainfall to make alternative or supplementary estimates^6–8^. Therefore evaluation of satellite-based precipitation products on quality and feasibility are quite necessary for the improvement of product quality and research of climate change.

As the Tropical Rainfall Measuring Mission (TRMM) satellite with the first space-borne precipitation radar has completed over 17 years’ operation since 1997^9^, its products have been widely applied to the fields of hydrological modeling^10,11^, meteorological drought^12,13^, agricultural science^14,15^ etc., and they have been evaluated with a better performance than rain gauges^16^. Actually, in recent years, differences between TRMM rainfall products and rain gauge observations have been analyzed across the world. Sealy, *et al*.^17^ found that the rain rates of TRMM 3B43 are lower than the ones of TRMM 3A25 among the wet season for West Africa and the Sahel. Fleming, *et al*.^18^ compared 3B43 rainfall product with the Australian Bureau of Meteorology regridding dataset, which shows a correlation coefficient of R = 0.93 between those two datasets. Wang, *et al*.^19^ pointed out that 3B43 data presents a great consistency with the ground-based measurements in autumn and fairly steady capacity during the relative arid and humid years in the Qinling-Daba Mountains of China. Curtarelli, *et al*.^20^ determined that there is a good agreement (*r* > 0.97) between the TRMM 3B43 monthly mean rainfall product and *in-situ* data over the Itumbiara Reservoir drainage area in Central Brazil but 3B43 tends to overestimate precipitation by approximately 1.24%. Tao, *et al*.^21^ disclosed that the best agreement of 3B43 precipitation data with observations tends to occur in autumn (SON) and large bias can be observed during spring^22^ and winter (DJF) in Jiangsu Province, China. Nastos, *et al*.^23^ concluded that the correlations between 3B43 gridded precipitation and ground based database in winter is very high (*r* > 0.90) for the entire Greek domain although 3B43 overestimates the precipitation over the Aegean and Ionian Sea, the coastal areas of Asia Minor and western Greece in autumn (>60 mm). Wolff and Fisher^24^ revealed that the TRMM 3G68 instantaneous rain intensities agree well with ground validation derived via radars and rain gauges in Florida except extreme heavy rain events are retrieved unsuccessfully. Buarque, *et al*.^25^ emphasized that uncertainty in rainfall characteristics is underestimated after compared four rainfall characteristics computed from the Brazilian rain gauge network and TRMM 3B42 datasets in the Amazon Basin. Haigen, *et al*.^26^ reported that a good linear relationship exists between 3B42 monthly precipitation and rain gauge rainfall data over the semi-humid Weihe River catchment of Yellow River Basin in China. Chen, *et al*.^27^ showed that 3B42 has good skill at detecting intense tropical cyclone rainfall as well as good correlation and pattern matching with the Comprehensive Pacific Rainfall Database observations. Chen, *et al*.^28^ also found that TRMM 3B42 rainfall data has a high precision and a good correlation with the observed precipitation in the Dongjiang River Basin amid Peral River Delta, China.

However, accuracy of the TRMM precipitation data depends on various factors, such as regions, seasons and spatial-temporal scales, etc. Liu^29^ examined TRMM 3B42 Version 6 and Version 7 3-hourly products on a global scale, which suggests that heavy rainfall estimates in Version 6 are larger than those in Version 7 throughout summer and winter for both land and oceans even though both versions display a good coincidence in heavy rain regimes. Khan, *et al*.^30^ stated that 3B42 Version 7 precipitation products, during the monsoon season, correlate highly with intense rain rates (>30 mm/day) whose bias is about ±20% while overestimating light rain rates with nearly 100% bias over mountain ranges in the Indus Basin of Pakistan. Li, *et al*.^31^ demonstrated that 3B42 daily rainfall data does not describe the occurrence and contribution rates of precipitation accurately, nevertheless, monthly ones have a good linear relationship with observed rainfall of rain gauges located at Poyang Lake Basin derived from Xinjiang catchment of China. Almazroui^32^ highlighted that TRMM 3B42 tends to overestimate rainfall over Saudi Arabia, particularly the coastal areas, although correlation coefficient between TRMM and observation is approximately 0.90 with a 99% level of significance on the monthly scale. Habib, *et al*.^33^ assessed that TRMM Multisatellite Precipitation Analysis (TMPA) products track the temporal evolution and fluctuations of surface rainfall on a storm scale fairly well with correlation values ranging from 0.50 to 0.80 and deviation degrees varying within ±25% in Louisiana, USA. Islam and Uyeda^34^ verified that 3B42 Version 5 data overestimate the rainfall before monsoon season in dry areas but underestimate it during the monsoon period in wet regions over Bangladesh. Condom, *et al*.^35^ found that TRMM 3B43 data overestimate the *in-situ* data between May and August whereas underestimating them in the period from October to March over the mountainous areas of the Peruvian Andes. Hashemi, *et al*.^36^ observed that temperature in the conterminous United States and the bias between 3B43 precipitation product and the ground-based gridded monthly precipitation dataset from the Climate Prediction Center have a weak linear relationship but a moderate inverse linear relationship appears between elevation and the bias. Thus, more studies concentrated on specific terrain and climate are indispensable in further understanding topographical and climatological impacts on TRMM datasets in the world.

The Yangtze River Delta (YRD) that accounts for 2.2% of China’s territory covers the area from 116.36°E to 122.95°E and 27.14°N to 35.13°N. This region suffers flooding disasters which cause heavy loss on property and society throughout the year^37^. Hence it is critically important to study its precipitation regime to make exact forecast. But until now, there are only few studies^38,39^ employing TRMM 3B42 data over the YRD area, the application of 3B43 dataset is rare too, not even to mention evaluating it and detecting impacts of topography and land use on it. So the objective of this paper is providing scientific references to the applicability of TRMM 3B43; to achieve this goal, the quality of their precipitation products is investigated on different spatial-temporal scales as well as their pertinence to observational data. Furthermore, topography might have impact on the backscatter direction of TRMM microwave antenna beam which can also be affected possibly by different underlyings/landcovers owing to their different backscattering and absorption properties of microwave. Hence, influences of elevation and landcover are also conducted in this paper.

## Data and Methods

### Study domain

**Figure 1:**
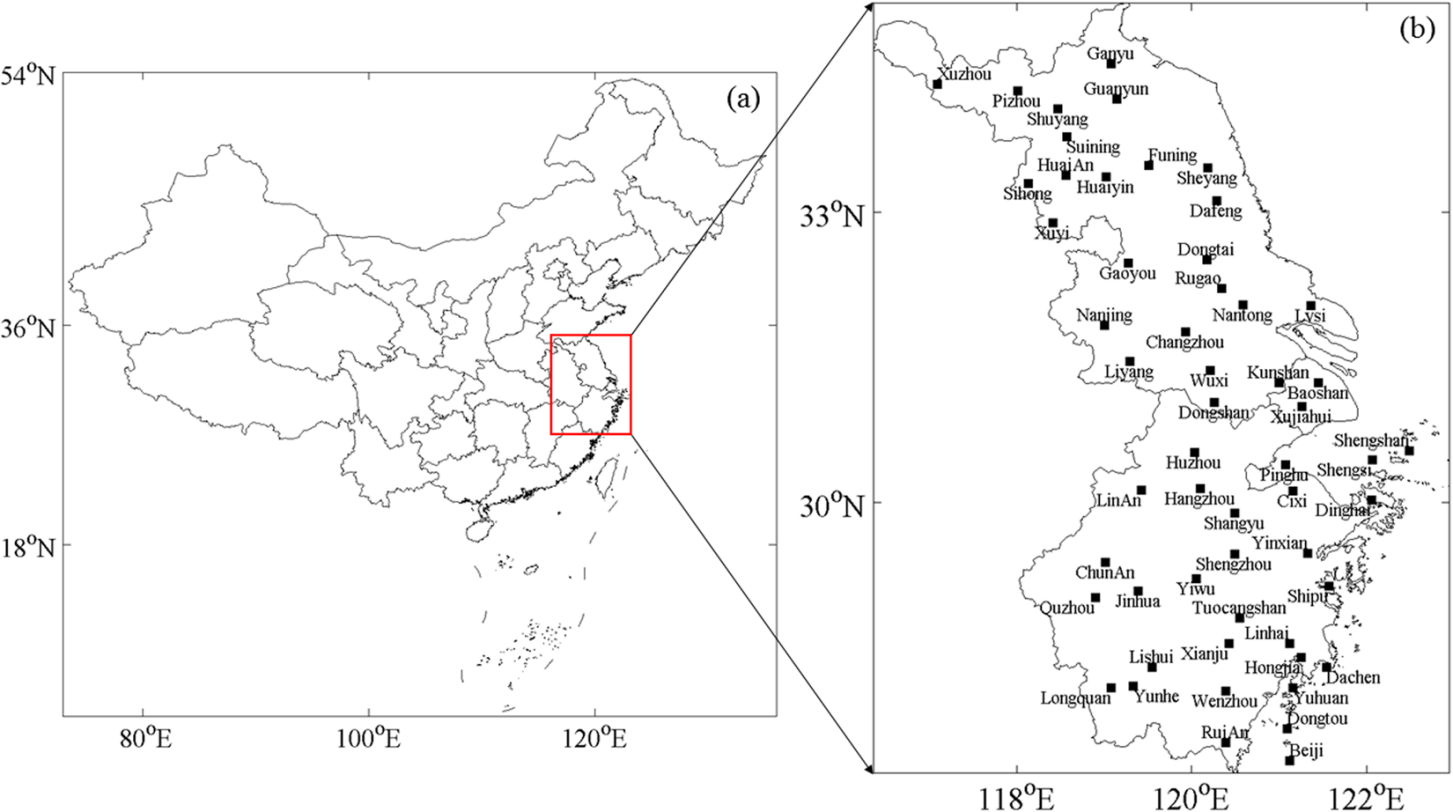
(**a**) Location of the Yangtze River Delta; (**b**) distribution of 56 meteorological stations. Jiangsu Province (116.36°E~122°E, 30.75°N~35.13°N) is situated in the northern Yangtze River Delta while Zhejiang Province (118°E~122.95°E, 27.14°N~31.5°N) in the southern part. Baoshan and Xujiahui are in the Shanghai Municipality (120.87°E~122.2°E, 30.67°N~31.88°N). Maps were generated by MATLAB R2014a (https://www.mathworks.com/products/new_products/release2014a.html).

The Yangtze River Delta, located in the east coast of China (Fig. 1a), covers about 99600 km^2^ with a population of 150 million. It includes 16 core cities (the Shanghai Municipality, 7 cities in the north of Zhejiang Province and 8 cities in the south of Jiangsu Province) and contributed 18.5% of the national GDP in 2014^40^.

The importance of this delta lies not only in its unique geographical position and complex geomorphology, but also in the function that it performs in the fields of regional water cycle, climate change and ecosystems as well as China’s economic and social development.

### Landcover and elevation

**Figure 2:**
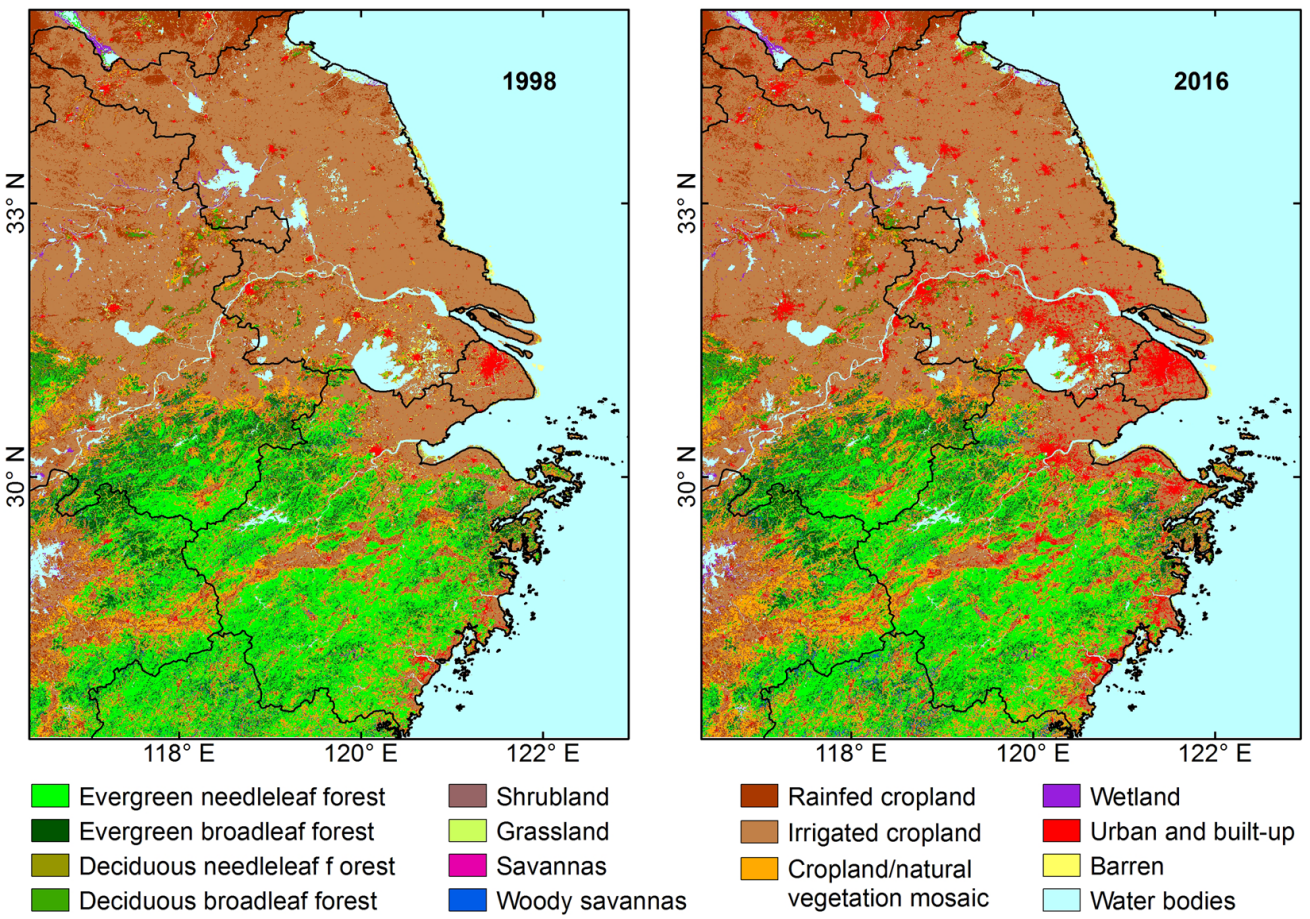
Land use map of the Yangtze River Delta^47^. Inland water bodies are all lakes while the one outside land is China East Sea. Figures were generated by ArcMap (http://desktop.arcgis.com/en/arcmap/).

Derived from the Land Long Term Data Record archive (http://ltdr.nascom.nasa.gov), Fig. 2 shows distinctly that the southern part of the YRD region is mainly occupied by the evergreen needleleaf forest while irrigated cropland and urban land are the major land type in Jiangsu Province and Shanghai municipality. Although urban and built-up land have expanded dramatically over the Yangtze River Delta from 1998 to 2016, the overall distribution of land use has not changed.

**Figure 3:**
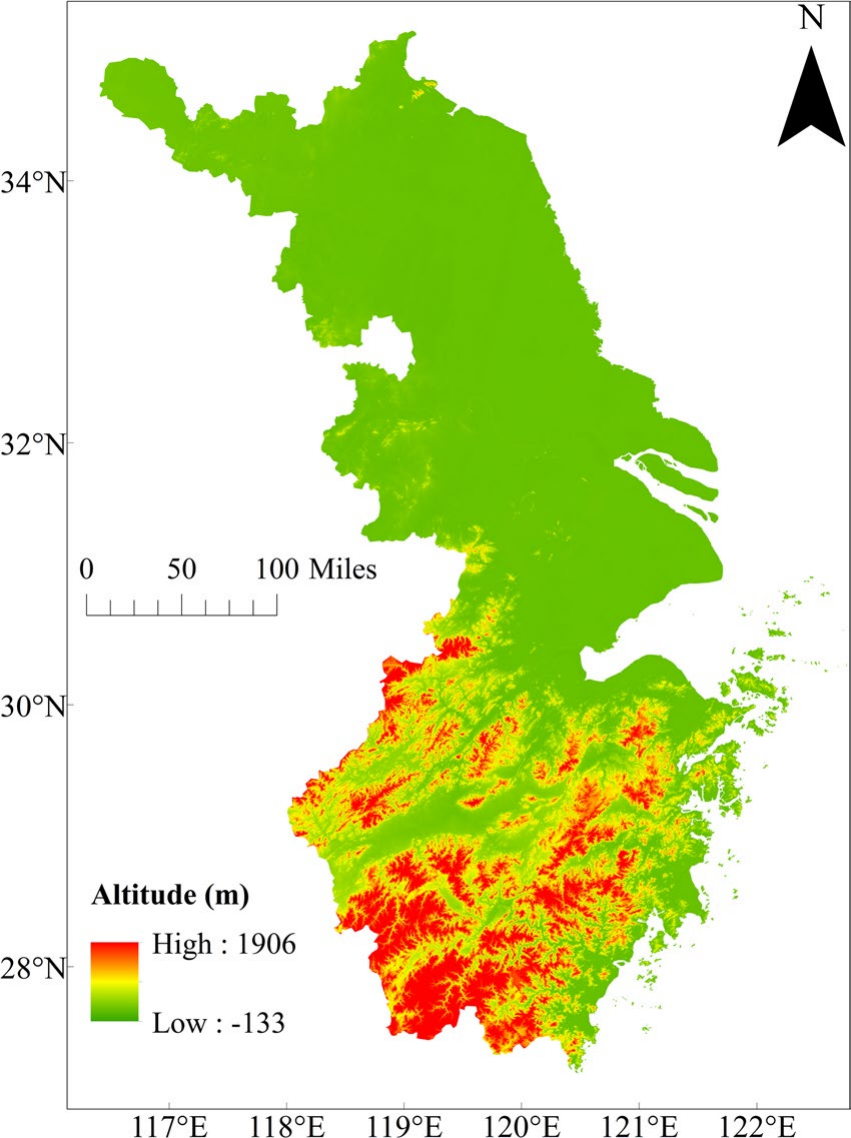
Topography of the Yangtze River Delta. The map was generated by ArcMap (http://desktop.arcgis.com/en/arcmap/).

Topography map (Fig. 3) that utilizes the Digital Elevation Model dataset with 30 arc seconds resolution (https://lta.cr.usgs.gov/GTOPO30) reveals the conspicuous elevation difference between the northern and southern YRD zone. Plains lower than 100 m are in Jiangsu and Shanghai municipality whereas mountains higher than 500 m spread over Zhejiang Province from 27.5°N to 30°N.

### TRMM 3B43 Version 7 data

Released as one of the TMPA products in July 2011, the latest TRMM 3B43 Version 7 data between 1998 and 2016, which were downloaded from the NASA Goddard Space Flight Center (https://pmm.nasa.gov/data-access/downloads/trmm), combine multiple independent precipitation estimates from the TRMM Microwave Imager (TMI), advanced microwave scanning radiometer for Earth Observing Systems and Global Precipitation Climatology Centre rain gauge analysis, etc^4^. All input microwave data starting in January 1998 are summed for month on quasi-global grids with spatial resolution of 0.25° (~25 km) for the latitude belt from 50°N to 50°S and combined with the gauge analysis using inverse-error-variance weighting to form the best-estimate precipitation rate and root-mean-square error estimates^41^. These gridded estimates have a monthly temporal resolution but a two-month latency. The annual 3B43 rainfall data were generated by accumulating all 12 monthly datasets.

### Rain gauge data

Monthly and annual rainfall data recorded by the tipping bucket rain gauges of 56 meteorological stations illustrated in Fig. 1b (24 in Jiangsu Province, 2 in Shanghai Municipality and 30 in Zhejiang Province) were obtained from the China Meteorological Data Service Center (https://data.cma.cn/) to represent the general precipitation scenario of the YRD area within the period of 1998~2016. All precipitation data in water bodies are measured upon islands which are tiny spots in maps on account of the whole resolution setting.

It is noteworthy that the rain gauges utilized in the observed precipitation may not be absolutely irrelevant to those used in 3B43 since data from some meteorological stations could also exist in TRMM datasets according to the China Meteorological Administration^2^.

### Statistical indices and validation method

To evaluate the performance of TRMM 3B43 data versus observational values, three validation statistical indices, including relative bias (hereafter Bias), correlation coefficient (hereafter CC) and root mean square error (hereafter RMSE), are chosen in this paper. They are defined by the following equations:

1 $${\rm{Bias}}=(\frac{{\sum }_{i=1}^{n}TRM{M}_{i}-{\sum }_{i=1}^{n}OB{S}_{i}}{{\sum }_{i=1}^{n}OB{S}_{i}})\times 100 \% $$

2 $${\rm{CC}}=\frac{{\sum }_{i=1}^{n}(OB{S}_{i}-\overline{OBS})\cdot (TRM{M}_{i}-\overline{TRMM})}{\sqrt{{\sum }_{i=1}^{n}{(OB{S}_{i}-\overline{OBS})}^{2}\cdot {\sum }_{i=1}^{n}{(TRM{M}_{i}-\overline{TRMM})}^{2}}}$$

3 $${\rm{RMSE}}=\sqrt{\frac{{\sum }_{i=1}^{n}{(OB{S}_{i}-TRM{M}_{i})}^{2}}{n}}$$

where *OBS*_*i*_ is the observed rainfall while *TRMM*_*i*_ is the value of 3B43; $\overline{OBS}$ denotes the precipitation mean of rain gauge station whereas $\overline{TRMM}$indicates the one of 3B43; *n* is the total sample size of monthly or annual precipitation in the analysis.

When compared with the pixel/grid measurements, *in-situ* measurements are limited on point scale with uneven distribution, actually one *in-situ* station usually covers about 3~4 pixels, therefore it is hard to acquire accurate and high-resolution data on global scale^19^, especially in inaccessible areas (mountains and oceans). Nonetheless, only *in-situ* measurements can exam the accuracy of pixel/grid data, which is still the essential part of evaluation on TRMM 3B43 dataset. For this purpose, based on meteorological station’s longitude and latitude, four closest pixels which represent the general rainfall status around every station in the Yangtze River Delta are selected. After being averaged, pixel values are used to calculate correlation coefficient and root mean square error of 3B43 annual and monthly precipitation products versus observational rainfall data in corresponding time scales, respectively.

## Results and Discussion

### Spatial and temporal distribution of precipitation

#### Annual precipitation

**Figure 4:**
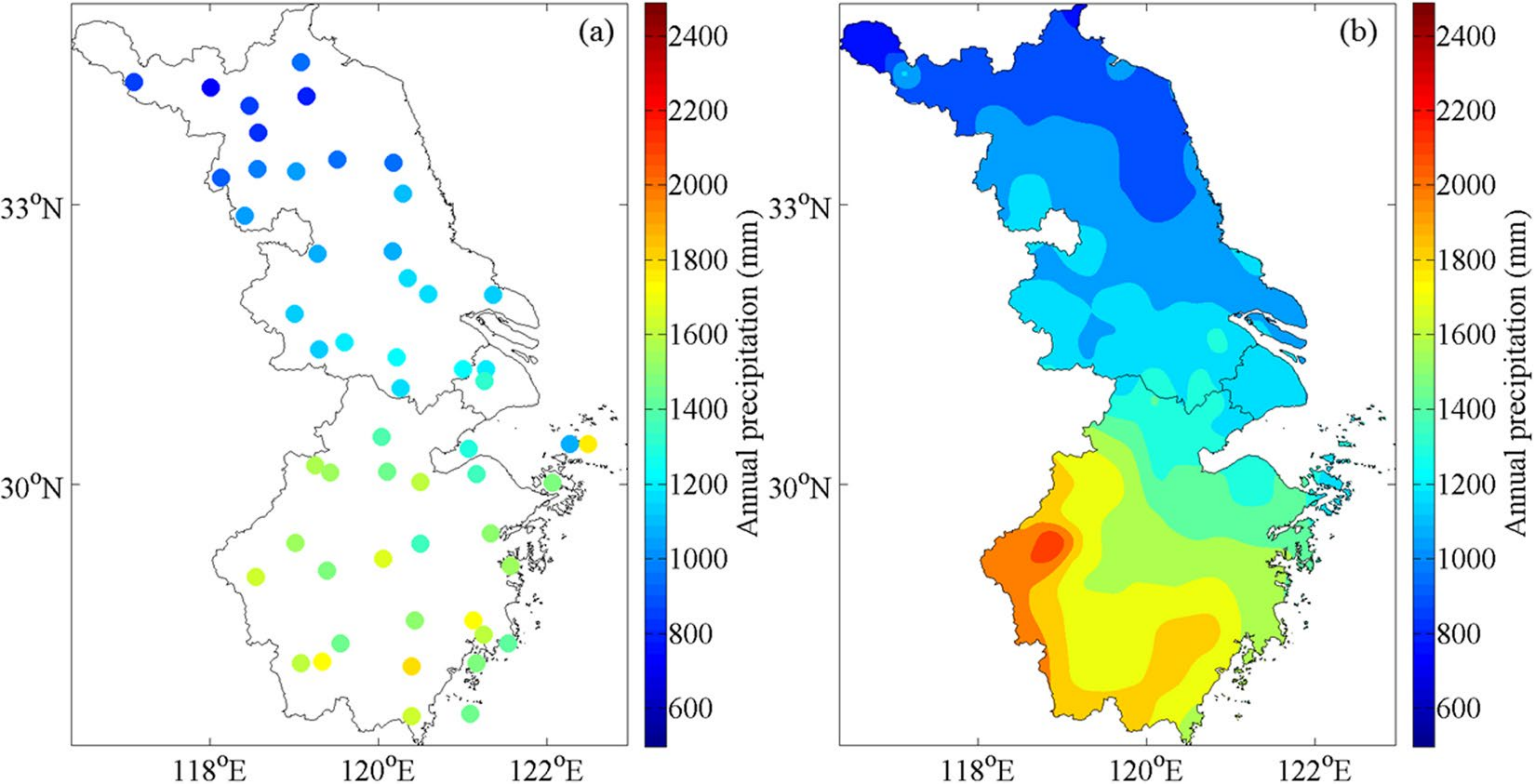
Spatial distribution of annual precipitation average over the Yangtze River Delta for (**a**) observational and (**b**) TRMM 3B43 data between 1998 and 2016. Maps were generated by MATLAB R2014a (https://www.mathworks.com/products/new_products/release2014a.html).

Annual precipitation is defined as the annual mean of precipitation from 1998 to 2016. As shown in Fig. 4, both observational and TRMM 3B43 data reflect the gradient distribution trend that annual precipitation increases progressively from northwest (<600 mm/year) to southwest (>1800 mm/year) of the YRD region except a remarkable precipitation center (>2200 mm/year) which is retrieved by TRMM possibly due to its higher spatial resolution appears around ChunAn (119.01°E, 29.37°N). In addition, the precipitation transition belt between northern and southern YRD region concentrates around the junction of Jiangsu Province, Shanghai Municipality and Zhejiang Province, extending along the administrative boundaries of Zhejiang and Jiangsu according to ground-based observation, while the one presented by TRMM has a wider scope with its “upper” limit expanding approximately 1° (~111 km) northward.

**Figure 5:**
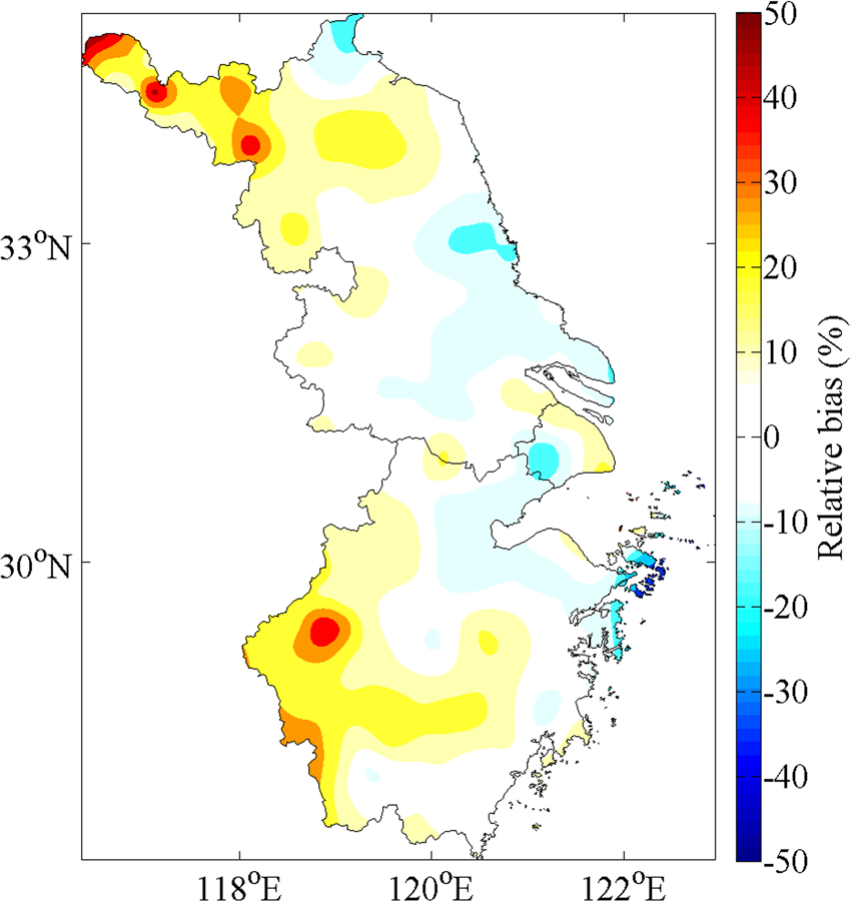
Relative bias of TRMM 3B43 estimates against the interpolated observational data for average annual precipitation over the Yangtze River Delta between 1998 and 2016. Maps were generated by MATLAB R2014a (https://www.mathworks.com/products/new_products/release2014a.html).

Spatial distribution of relative bias (Fig. 5) reveals that 3B43 estimates the annual rainfall at most parts of the Yangtze River Delta with the best performance (Bias varies from −10% to 10%), which is corresponding to the highest rate (57.59%) in Table 1. However, in spite of accounting for only 2.26% among all grids, three anomaly centers with Bias exceeding 30% lie near Xuzhou (117.09°E, 34.17°N), Shuyang (118.47°E, 34.05°N) and ChunAn, respectively. Nevertheless, we need to emphasize that the emergence of anomaly doesn’t mean TRMM fails to retrieve precipitation in the above zones; on the contrary, it could be attributed to the difference between high-precision grids of 3B43 and sparse station network, leading to detailed precipitation found by the satellite rather than rain gauges, which is manifested as anomaly in the map, but the exact reason must be further investigated.

**Table 1:** Bias distribution for TRMM 3B43 annual rainfall estimates against observational data in the Yangtze River Delta.

Bias (%)	**<**−**50**	−**50~**−**30**	−**30~**−**10**	−**10~10**	**10~30**	**30~50**	**≥50**
Rate	0.00%	0.08%	1.19%	57.59%	38.88%	2.24%	0.02%

Overall, TRMM 3B43 tends to overestimate annual precipitation over the YRD area as relative bias ranging within 10~30% has an nonnegligible proportion — 38.88% (Table 1) although several negative Bias centers exist alongside the coastal zones, e.g. Dafeng (120.29°E, 33.12°N), Xujiahui (121.26°E, 31.12°N), etc., which are constituted of 1.19% grids whose Bias are between −30% and −10%. Figure 5 also displays that relative bias increase inland from negative values on coastland to positive values in hinterland of the Yangtze River Delta as well as absolute values of them, which is another proof of rainfall overestimation from 3B43 because the overestimated effects brought by positive Bias outdo the underestimation of negative ones.

#### Monthly precipitation

**Figure 6:**
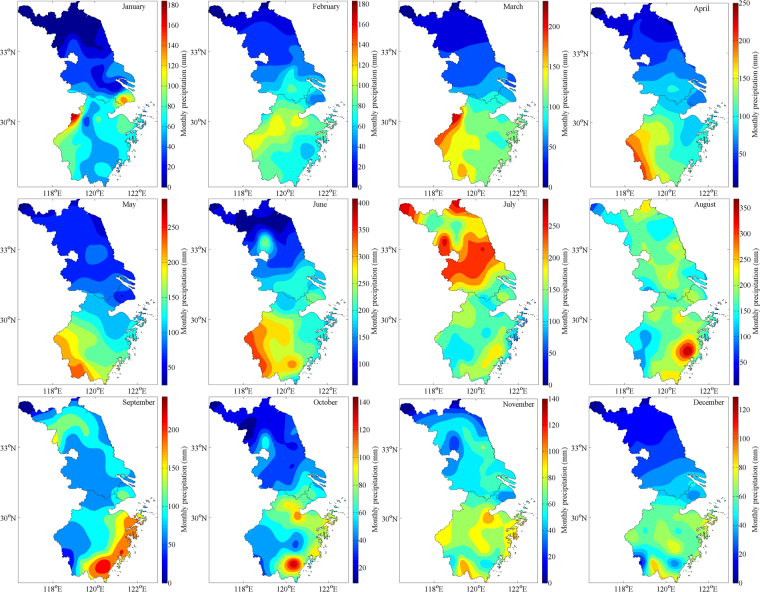
Spatial distribution of interpolated observational monthly mean precipitation from January to December between 1998 and 2016. Maps were generated by MATLAB R2014a (https://www.mathworks.com/products/new_products/release2014a.html).

**Figure 7:**
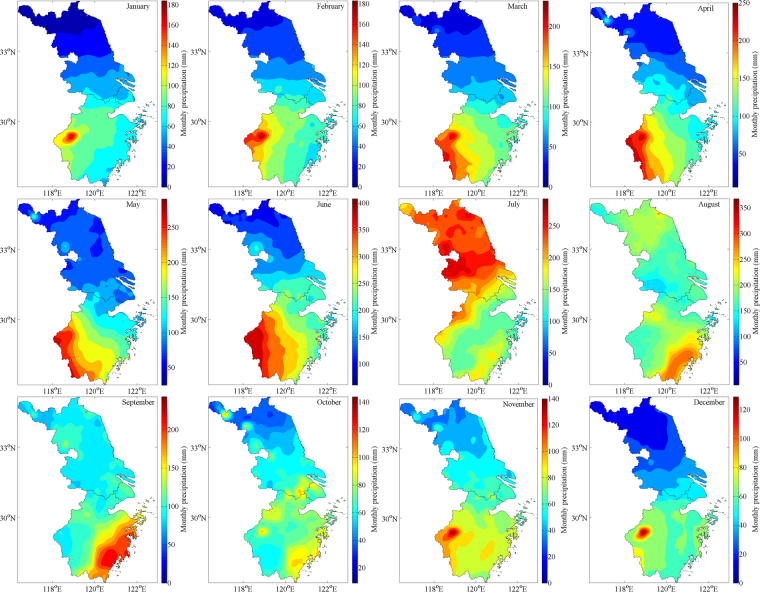
Spatial distribution of monthly mean precipitation for TRMM 3B43 from January to December between 1998 and 2016. Maps were generated by MATLAB R2014a (https://www.mathworks.com/products/new_products/release2014a.html).

Figures 6 and 7 illustrate that interpolated observational data and TRMM 3B43, especially in April, May, June and September, share the similar spatial precipitation pattern that is rainfall amount in the southern YRD region is more than the northern one during the period of January to December excluding the inverse occasion happened in July because of the westward extension and northward jump of Western Pacific Subtropical High (WPSH)^42,43^, resulting in rainband’s pushing north and widespread heavy rain, thereby precipitation of the Yangtze River Delta enhances totally. Furthermore, after combining two datasets together, we find the spatial-temporal variation of monthly precipitation could be concluded as the following pattern: from January to June, the precipitation center around ChunAn deepens and expands gradually until occupying the southwestern part of Zhejiang Province and stretching across 2° (~222 km) in latitude, 1° in longitude (Fig. 7); after abrupt shift of rain belt in July, a short-lived precipitation center forms near Linhai (121.12°E, 28.52°N) in August, develops and takes up the whole southeastern coast of Zhejiang in September and perishes in October (Fig. 6); in the next two months, ChunAn Center appears again and the above process starts to cycle.

**Figure 8:**
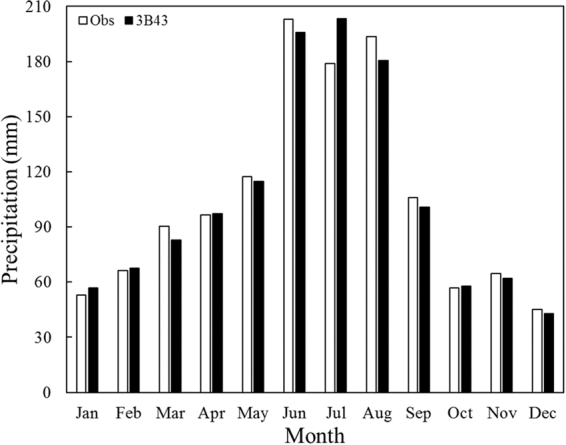
Variation of monthly precipitation average for observational and TRMM 3B43 data over the Yangtze River Delta between 1998 and 2016.

In terms of the monthly mean precipitation comparison between observed and 3B43 results (Fig. 8), it is salient that rainfall amount over the Yangtze River Delta, resulted from its whole rising caused by the movement of WPSH, is sharply large (almost 200 mm/month) in summer (June, July and August), before which it experiences a steady and continuous growth that is in accord with the developmental tendency of ChunAn precipitation center, bringing about abundant precipitation in the YRD region till peaking at 120 mm/month in May. Besides, the fluctuant descent within September and November just coincides with the extinction of Linhai precipitation center whose manifestation is monthly rainfall amount dropping off dramatically from the maximum (about 110 mm) to the minimum (60 mm), and resurgence of ChunAn Center, embodied as a slight ascent of precipitation.

**Figure 9:**
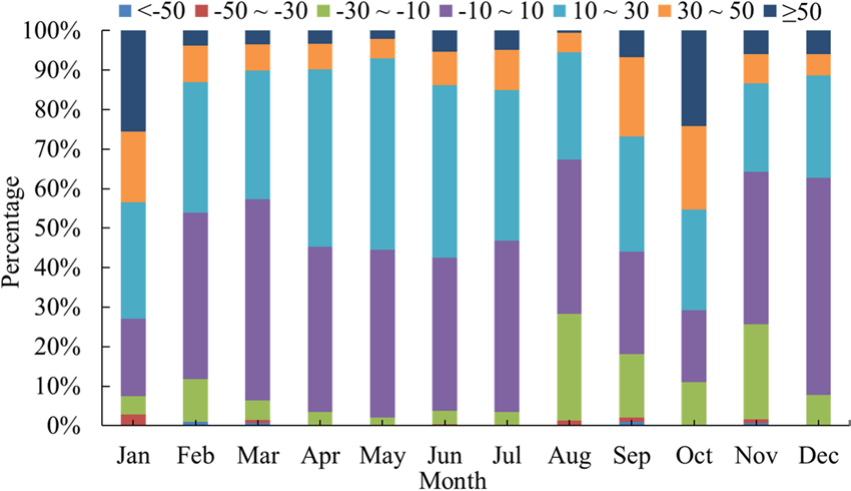
Bias distribution for TRMM 3B43 monthly rainfall estimates against observational data in the Yangtze River Delta.

In general, even though TRMM 3B43 overestimates monthly precipitation in the YRD area, one typical illustration is 3B43 deviating from the observed data to the greatest extent (13.69%) in July (Fig. 8), there are still several underestimation conditions, whose cause is that overestimated impact of positive relative bias could be offset by the negative ones on account of the higher absolute values of the latter in the averaging process. This could also explain why monthly precipitation means derived from TRMM agree well with the observational ones in Fig. 8 while Bias distributed between positive intervals of Fig. 9 have overwhelming percentages in some months, e.g. February, April and October.

### Accuracy of precipitation

**Figure 10:**
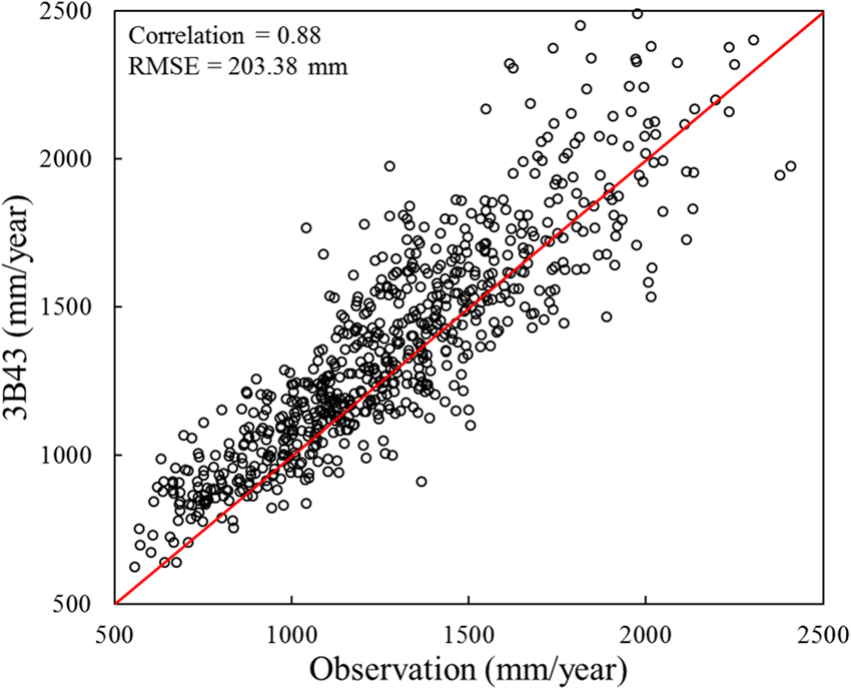
Scatterplots of annual precipitation recorded by TRMM 3B43 products and gauge stations over the Yangtze River Delta.

According to the validation method in 2.5, TRMM grid data are evaluated on accuracy and results are displayed in Fig. 10 and Table 2. For rainfall amount on year time basis, the CC between 3B43 and rain gauges is 0.88 (statistically significant at the 99% confidence level) and the RMSE is 203.38 mm. Points have the inclination to aggregate around 1200 mm/year at which higher correlation comes out. Moreover, there are more points located above the red line in 45° angle than the ones below it, which means, like we summarized in 3.1.1, the 3B43 results incline to overestimate annual precipitation in the YRD region.

**Table 2:** CC and RMSE of monthly precipitation recorded by TRMM 3B43 products and gauge stations over the Yangtze River Delta.

	Jan	Feb	Mar	Apr	May	Jun	Jul	Aug	Sep	Oct	Nov	Dec
CC	0.95	0.93	0.96	0.92	0.91	0.90	0.84	0.79	0.82	0.90	0.93	0.95
RMSE (mm)	17.26	17.39	19.61	26.17	32.17	58.55	59.33	66.15	49.29	29.03	19.79	13.95

On the other hand, in the area of time on month scale, CC reaches the highest point at 0.96 in March and the lowest point at 0.79 in August, meanwhile the second maximum (0.95) in December and January is notable, too. All of them depict the variation of pertinence between 3B43 data and observed ones, i.e., declining from the summit (winter and spring) to the nadir of summer, which is also applied to the changing trend of precision although RMSE whose higher value means lower precision varies in the opposite direction: bottoming out at 13.95 mm in December and peaking at 66.15 mm in August.

### Impact of elevation on precipitation

**Figure 11:**
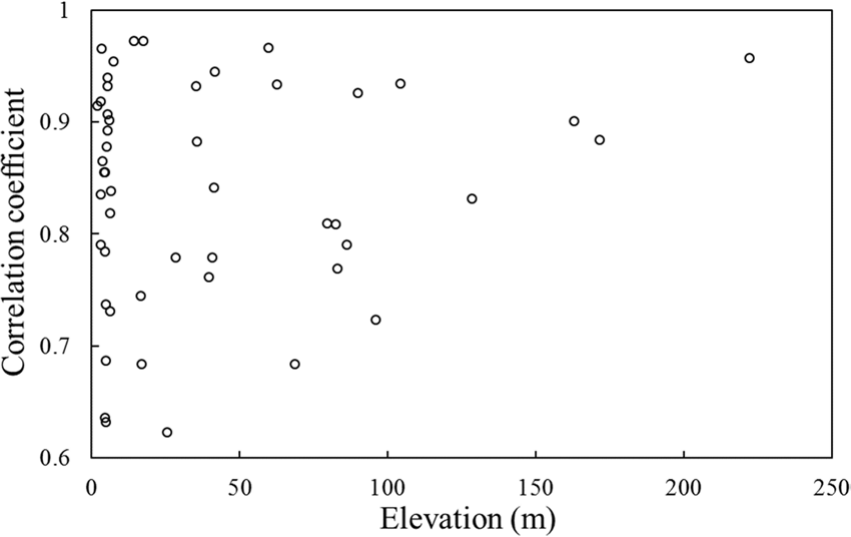
Correlativity between elevation and correlation coefficient of TRMM 3B43 precipitation versus observed data on annual scale.

Considering the distinct topography of the Yangtze River Delta that may cause terrain-induced errors on remote sensing retrievals^30^, finding the effects of it on quality of 3B43 products is indispensable. However, there are no obvious relationships between CC and elevation (Fig. 11).

A possible explanation to this phenomenon is that climatological calibrations of 3B43 eliminate topographical influences during the adjustment process^44^ so that 3B43 products are not sensitive to terrain’s enhancement. In fact, according to precursory studies^2,30,32^, only at regions with higher elevation (>1000 m) but lower rainfall amount (<200 mm/year) does topography present a slight linear relationship with daily TMPA products, none of which has something to do with the circumstances discussed in this paper. In fact, statistical indices, including correlation coefficient, perform better in the low-altitude areas receiving high intensity of rainfall, which fits perfectly to the YRD region of our study.

### Impact of landcover on precipitation

Previous studies^22,45^ have investigated the characteristics of TRMM precipitation measurements for different landcovers in Africa and Indo-China Peninsula. Considering different regions have different underlyings as well as impacts on TRMM precipitation products, we classify the YRD zone into four land types and calculate CC, RMSE and Bias of 3B43 annual rainfall products against observational data over each particular type of landcover. Some studies^22,45,46^ have proved that backscattering coefficients observed by TRMM microwave imager/scatterometer are sensitive to the surface roughness, rougher underlyings have smaller backscattering coefficients, which is beneficial to the precipitation retrieval algorithm of TRMM that contains estimates from the TMI^41^. So that’s why in Table 3, 3B43 has stronger correlativity with rain gauge data and higher precision over cropland and urban. Forest and water produce larger backscattering coefficients which lower the estimation accuracy of TRMM.

**Table 3:** CC and RMSE of annual precipitation recorded by TRMM 3B43 products and gauge stations over different landcovers in the Yangtze River Delta.

	**Forest**	**Water**	**Cropland**	**Urban**
**CC**	0.76	0.78	0.89	0.91
**RMSE (mm)**	256.32	226.71	154.93	129.33

Additionally, urban and cropland’s higher rates of best performance in Table 4 demonstrate the higher precision of 3B43 upon these two types of landcover. But for all four land types, TRMM has the inclination to overestimate the rainfall in the Yangtze River Delta because of the dominated proportion of positive Bias.

**Table 4:** Bias distribution for TRMM 3B43 annual rainfall estimates against observational data over different landcovers in the Yangtze River Delta.

Bias (%)	Forest	Water	Cropland	Urban
<−50	0.00%	0.00%	0.00%	0.00%
−50~−30	0.41%	0.00%	0.40%	0.00%
−30~−10	10.79%	11.11%	7.69%	4.63%
−10~0	21.16%	13.89%	20.65%	25.93%
0~10	26.14%	25.93%	31.98%	35.19%
10~30	35.27%	39.81%	31.98%	29.63%
30~50	5.39%	7.41%	6.48%	4.63%
≥50	0.83%	1.85%	0.81%	0.00%

## Conclusions

Aiming to evaluate feasibility of TRMM 3B43 products over the Yangtze River Delta, this paper exploited rain gauge data of 56 meteorological stations from 1998 to 2016 to contrast. The primary results are listed below.

Annual precipitation, retrieved by 3B43 and observed data, both distribute in a southward gradual growth pattern that begins from northwestern area with less than 600 mm/year but surpasses 1800 mm/year in the southwest. With Bias exceeding 30%, ChunAn precipitation center (119.01°E, 29.37°N) where rainfall amount is more than 2200 mm/year, Xuzhou (117.09°E, 34.17°N) and Shuyang (118.47°E, 34.05°N) anomaly centers are only discovered by 3B43 data. Besides, 3B43 results perform the best over the majority of the YRD region because Bias lying within the interval of −10~10% have the largest ratio — 57.59%; and tend to overestimate precipitation as 41.14% Bias are larger than 10% although there are some negative value centers located near the coastal zones, such as Dafeng (120.29°E, 33.12°N), Xujiahui (121.26°E, 31.12°N), etc.

Compared with grid-interpolated observational data, the TRMM 3B43 overestimates monthly precipitation overall, especially by the biggest degree — 13.69% in July. The spatial distribution that southern rainfall amount is more than the north is all manifested among twelve months apart from exceptional northward movement of rain belt caused by the westward and northward expansion of Western Pacific Subtropical High (WPSH) in July, which drastically enriches the precipitation of entire YRD zone and drives it up to the peaking point at 200 mm/month. Before summertime (JJA), Zhejiang Province experiences the developing process of ChunAn center until occupying its southwestern 24642 km^2^ territory roughly, which brings a stable and unremitting increase of monthly rainfall amount of the YRD region till arriving at the maximal 120 mm/month in May; on the other hand, after JJA, the transitory Linhai precipitation center (121.12°E, 28.52°N) reaches its heyday in September over the whole southeastern coastland of Zhejiang but vanishes promptly in October, which coincides with a plummeting of rainfall quantity from 110 mm/month to the minimal 60 mm/month, followed by a modest enhancement in November which indicates that ChunAn center revives and monthly precipitation series come into circulation. Contrary to the annual situation, TRMM’s overestimated effect on monthly precipitation could be neutralized by negative Bias as their absolute values are higher when calculating means though positive ones have unparalleled percent in February, April and October.

Yearly rainfall’s CC and RMSE of 3B43 products against ground-based observed data are 0.88 and 203.38 mm, respectively. Stronger correlationship emerges at about 1200 mm/year. CC for monthly scale arrives at the top (0.95) in winter but sinks to the bottom (0.79) in summer. That tendency adapts to the precision as well while RMSE whose lower value means higher precision falls to the nadir (13.95 mm) in December but climbs to the zenith (66.15 mm) in August. For the annual precipitation scatterplot of 3B43 with respect to observations, scattered points are more likely to float above the 45° diagonal line of the graph implying TRMM’s overestimation at the Yangtze River Delta once again.

No conspicuous influences of elevation on CC is discovered in annual scale perhaps due to climatological adjustments of 3B43 removed it. Annual products reflect precipitation upon the urban and cropland better because their rougher surfaces have smaller backscattering coefficients.
